# Tomato Multi-Angle Multi-Pose Dataset for Fine-Grained Phenotyping

**DOI:** 10.1038/s41597-026-06926-9

**Published:** 2026-02-28

**Authors:** Yujie Zhang, Sabine Struckmeyer, Andreas Kolb, Sven Reichardt

**Affiliations:** 1 Institute for Breeding Research on Horticultural Crops, Julius Kuehn-Institute, Erwin-Baur-Street 27, Quedlinburg, 06484 Saxony-Anhalt Germany; 2 Computer Graphics Group, Center for Sensor Systems (ZESS), University of Siegen, 57076 Siegen, Germany

**Keywords:** Image processing, Data acquisition, Agriculture, Plant breeding, Machine learning

## Abstract

Observer bias and inconsistencies in traditional plant phenotyping methods limit the accuracy and reproducibility of fine-grained plant analysis. To address these limitations, TomatoMAP is introduced as a comprehensive dataset for *Solanum lycopersicum*. The dataset contains 68,080 RGB images: 3,616 high-resolution macrophotographs (3648 × 5472) with semantic annotations, and 64,464 moderate-resolution images (1080 × 1440) captured from 12 plant poses at four camera elevations. Each image is accompanied by manually annotated bounding boxes for seven regions of interest (leaves, panicle, flower clusters, fruit clusters, axillary shoot, shoot, and whole-plant area) and by labels spanning 50 BBCH classes representing phenologically growth stages. A general cascading structure is proposed. For real-time applicability, models emphasizing the accuracy-efficiency trade-off (MobileNetv3, YOLOv11, and Mask R-CNN) are prioritized and benchmarked against multiple state-of-the-art models. Performance is assessed using accuracy, mAP, inference FPS, and normalized confusion matrices. In a study involving five domain experts, AI models trained on TomatoMAP achieves comparable accuracy levels. Reliability of automated fine-grained phenotyping is supported by Cohen’s Kappa statistics and inter-rater agreement heatmaps.

## Background and Summary

*Solanum lycopersicum* (tomato), originating in the Andean region, constitutes a crop of agronomic and economic importance. Following its introduction to Europe in the 16th century, it has reached global dissemination and is now widely cultivated in various agroecological zones^1^. Worldwide, *S. lycopersicum* is important in the food supply chain and has significant implications for developmental biology, stress biology, and food science^2^. In 2024, the total production of *S. lycopersicum* for fresh consumption in Europe is 6,671,000 tonnes^3^. Moreover, *S. lycopersicum* has been firmly established as a model organism within the *Solanaceae* family and is widely recognized in the field of plant physiology^4^. This status is based on the availability of the fully sequenced and annotated genome along with an extensive repository of genetics, physiological and biochemical data^5^.

As a widely studied model plant, the accurate and high-resolution phenotyping is crucial for hypothesis-driven inquiries and the selection of agronomically desirable genotypes based on traits such as fruit quality, plant architecture, and disease resistance^2,6^. It also supports the dissection of complex trait architectures and advances marker-assisted and genomic selection, as well as the evaluation of responses to abiotic stresses, including drought, heat, and nutrient deficiencies^7^. Additionally, *S. lycopersicum* exhibits a remarkable degree of phenotypic plasticity^8^, reflected in the extensive variability observed across several agronomical important traits such as fruit size and coloration, flower development, leaf morphology, and sympodial shoot structure^9^. For example, the variation in fruit coloration serves as an optimal trait that provides an ideal feature for developing algorithms capable of accurate color segmentation, the diversity in fruit size and flower stage offers a resource for researching statistics models focused on robust size recognition and classification^10^. This phenotypic diversity provides exceptionally rich and varied scientific information for research aimed at improving fruit productivity, fruit quality^11^, and resilience to environmental stress^12^.

Due to subtle phenotypic variation in *S. lycopersicum*, advanced imaging and AI-driven phenotyping are essential for reducing observer bias (see Supplementary Fig. 1), enhancing data accuracy, and expediting breeding through high-throughput, standardized datasets. In recent years, there has been growing interest and demand in using artificial intelligence (AI) to automate the phenotyping process, as traditional methods are often labor intensive, subjective, and unable to scale for large datasets^13^. For example, Tian *et al*.^14^ reported a lightweight detection method for real-time tomato monitoring using edge computing devices and modified different structures of the “You Only Look Once” (YOLO) model. Lee *et al*.^15^ proposed a clip-type IoT camera based tomato phenotyping system for tracking and detecting tomato flowers and fruits using convolutional neural network (CNN). Rahman *et al*.^16^ presented a Bayesian network based context estimation algorithm for tomato growth phenotyping combining with CNN. Islam and Hatou^17^ established an AI-assisted five-stage tomato growth monitoring system based on CNN. Statistically, Baar *et al*.^18^ predicted fruit growth based on tomato diameter from 2D image and environment data with the sigmoid function and its extensions.

Monitoring data acquisition is integral to research involving *S. lycopersicum*. This species presents several advantages for the generation of high-resolution, fine-grained scientific datasets. Although structures such as buds, flowers, and axillary shoots may be smaller and less visually distinct, tomato plants remain favorable for imaging under controlled conditions. This can be attributed to the complex structure of their compound leaves^19^ and the agronomic characteristics of fruits^20^, which offer clear features. In recent years, numerous stable and reliable datasets have been published, contributing to plant phenotpying research, shown in Table 1.

**Table 1:** Comparison of published image datasets for tomato research.

Dataset Name	Author	Research Objects	Number of Images (in total)	Number of Instances (by annotation)	Categories (in total)
Tomato-Village^21,22^	Gehlot *et al*., 2023	Leaf Disease	5,067	166,886	8
TomatoOD^23,24^	Tsironis *et al*., 2020	Fruit Growth	277	2,418	3
PlantVillage^25–27^	Hughes and Salathe, 2015	Leaf Disease	54,306	54,306	38
Laboro Tomato^28^	Trigubenko, 2020	Fruit Growth	804	9,777	6
Tian, Kai Tomato Leaves^29,30^	Tian *et al*., 2022	Leaf Disease	1,000	1,000	3
Taiwan Tomato Leaves^31^	Huang and Chang, 2020	Leaf Disease	4,976	4,976	6
Maharashtra “Tomato Leaf”^32^	Khan *et al*., 2020	Leaf Disease	106	106	5
**TomatoMAP**		**Fine-Grained**	**68,080**	**720,938**	**67**

The “Tomato-Village” dataset^21,22^ comprises 5,067 images with subsets for disease detection (variation (a)), multi-class classification (variation (b)), and multi-label classification (variation (c)) tasks. Another notable dataset is the “TomatoOD”^23,24^, which contains 277 images with 2,418 annotated tomato fruit samples of unripe, semi-ripe and fully-ripe classes, enabling the tomato fruit localization and growth stage classification. A published large-scale datasets like the “PlantVillage”^25–27^ contain 54,306 classified instances with 38 classes focuses on leaf disease classification and detection, and the “Laboro Tomato” dataset^28^ contains 804 images of different tomato growth stages for detection and instance segmentation tasks. Kai Tian *et al*. published a tomato leaf disease dataset and trained three different deep learning network architectures^29,30^. Mei-Ling Huang *et al*. proposed a dataset “Taiwan tomato leaves” with data augmentation technique^31^. Saiqa Khan *et al*. presented “Tomato Leaf Dataset” generated with different mobile camera models for leaf disease detection purpose^32^.

However, most existing datasets contain single-pose, single-angle images with varying objects and low average labeled instances per class, limiting their use for high-accuracy model training and restricting analysis to 2D spatial features, see Table 2.

**Table 2:** Statistics of published image datasets for tomato research.

Dataset Name	Multi-View Information	Classification	Detection	Semantic Segmentation	Instance Segmentation
Tomato-Village^21^^22^	×	*✓*	*✓*	×	×
TomatoOD^23^^24^	×	*✓*	*✓*	×	×
PlantVillage^25–27^	×	*✓*	*✓*	×	×
Laboro Tomato^28^	×	×	*✓*	*✓*	*✓*
Tian, Kai Tomato Leaves^29,30^	×	*✓*	×	*✓*	×
Taiwan Tomato Leaves^31^	×	*✓*	×	*✓*	×
Maharashtra “Tomato Leaf”^32^	×	*✓*	×	×	×
**TomatoMAP**	*✓*	*✓*	*✓*	*✓*	*✓*

In contrast, multi-view information demonstrates significant potential. It facilitates the comprehensive characterization of the 3D phenotypic traits of *S. lycopersicum*^33,34^, preserving critical structural features such as interleaf distances, stem-leaf angles, and shooting topology. Traditional phenotyping faces challenges such as bias, labor intensity, and inefficiency^35^, especially under complex greenhouse conditions with limited class instances and no 3D structure^34^. To address these limitations, TomatoMAP is introduced as a fine-grained, multi-pose, multi-angle time-series dataset of *S. lycopersicum*. The resource comprises three subsets: (i) TomatoMAP-Cls for BBCH stage classification^36,37^, (ii) TomatoMAP-Det for object detection across key botanical regions of interest (ROIs), and (iii) TomatoMAP-Seg for pixel-wise semantic and instance segmentation.

## Methods

### Data Acquisition System and Data Generation

**Fig. 1:**
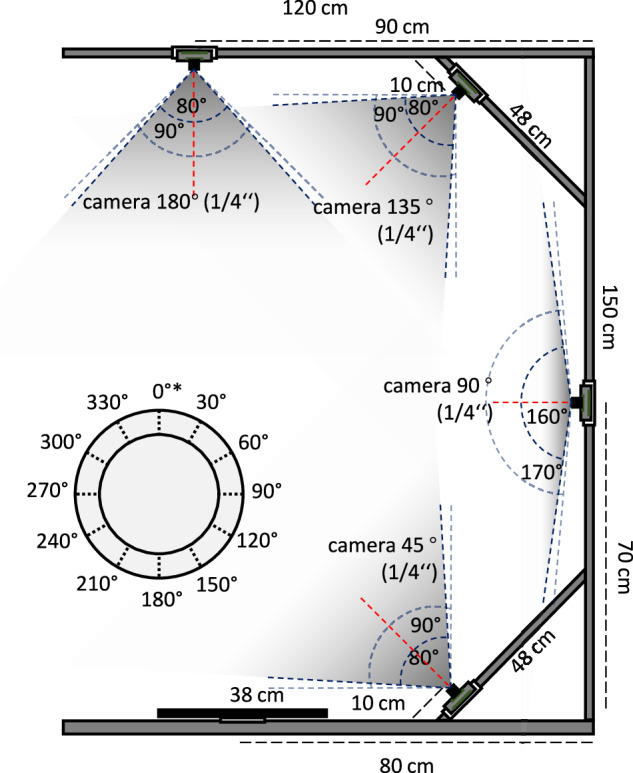
Data acquisition system, using an imaging array comprising four camera modules. The array provides a composite field of view, incorporating three modules with a 90° diagonal field of view and one module with a 170° diagonal field of view, positioned at vertical inclination angles of 45°, 90°, 135°, and 180° respectively. A turntable with 30° rotational increments is used for multi-pose purpose. To ensure consistent imaging poses for the same plant at different time points, an initial imaging pose position is marked on the tray (denoted as “*” in the figure).

Plant samples are cultivated under standardized conditions (see Supplementary Information 1). To enable multi-angle multi-pose imaging of *S. lycopersicum*, a scalable data acquisition station is first developed. It integrates a synchronized multi-camera array with a rotational platform, facilitating systematic and repeatable image capture across both spatial and temporal dimensions, see Fig. 1.

The imaging station comprises four OV5647 color CMOS 5-megapixel image sensors: three are equipped with 90^°^ lenses and one is equipped with a 170^°^ fisheye lens. These cameras are mounted at vertical inclination angles of 45^°^, 90^°^, 135^°^, and 180^°^, each with manual focus and an aperture of f/2.2. The three 90^°^ lenses provide fields of view of 90^°^ (diagonal) and 72^°^ (horizontal), while the 170^°^ fisheye lens offers ultra-wide 170^°^ (diagonal) coverage, allowing comprehensive coverage of the entire plant structure at close range (≈80*cm*)^38^.

**Fig. 2:**
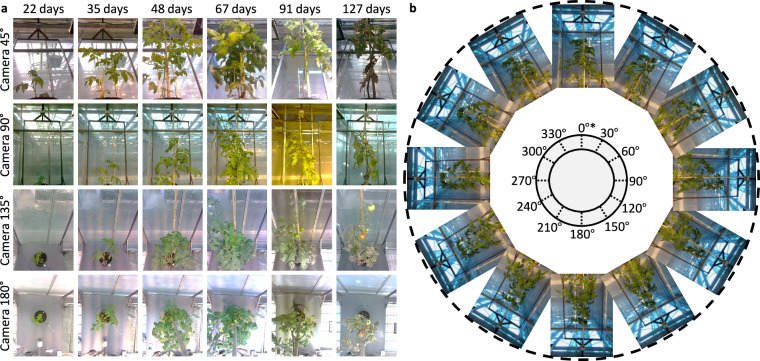
Dataset overview. **(a)** Multi-angle time series protocol of 101 plants subjected to varying lighting conditions within a greenhouse environment. Images are captured by cameras with a vertical inclination angles of 45°, 90°, 135°, and 180°, including different time points covering *S. lycopersicum* growth stages. **(b)** Multi-pose protocol utilizing a turntable. Sequential imaging of plant poses is achieved by every 30°. A plastic tray with marked initial point (as “*” in figure) is mounted on the turntable.

In total, the modest resolution subset contains phenotypic imaging data gathered over a 163-day period, from August to January. Data acquisition is performed with irregular intervals ranging from 1 to 13 days to adapt to different tomato growth rates and growth dynamics. The dataset comprises images from 101 individual plants, with a total of 32 acquisition time points. At each acquisition time point, plants are placed on the turntable and imaged at 30^°^ rotational increments, resulting in a comprehensive 360^°^ visual dataset. This yields 48 images per plant at each time point, covering multiple poses and angles. All camera modules record images at a resolution of 1080 × 1440 pixels and are synchronized to capture frames at each rotational step (see Fig. 2). This configuration ensures consistent sparse posed multi-view imaging, showing potential for 3D reconstruction and high-fidelity morphological analysis. Using this data acquisition system, a total of 64,464 images are captured and annotated for classification (TomatoMAP-Cls) and object detection (TomatoMAP-Det). The image and annotation files are formatted as Supplementary Information 2. Camera calibration is conducted using a planar chessboard pattern to estimate intrinsic and extrinsic parameters, including lens distortion coefficients. Calibration relied on the detection of 2D corner points and the formation of 3D-to-2D correspondences, following established photogrammetric methods^39^.

For semantic and instance segmentation tasks (TomatoMAP-Seg), 3,616 high-resolution macrophotographs are acquired using a Panasonic Lumix DMC-FZ1000 digital camera (Kadoma, Japan) at 3648 × 5472 pixel resolution.

### Annotation for Classification, Detection, and Segmentation

For labeling objectives, multiple annotation techniques are employed in parallel to support different computer vision tasks, including image classification, object detection, and semantic and instance segmentation. A comprehensive overview of the annotated categories and the corresponding number of labeled instances is presented in Table 3.

**Table 3:** Labeled instances and fine-grained class summary of TomatoMAP.

a) Classification & Detection Label Information		
Task	Fine-Grained Class	Instance Number
Classification	60 ≤ BBCH < 70	10,560
	70 ≤ BBCH < 80	29,328
	80 ≤ BBCH < 90	91,120
Detection	whole plant	64,626
	leaf	402,763
	flower clusters	23,127
	fruit clusters	37,684
	panicle	53,137
	axillary shoot	1,761
	shoot	319

b) Segmentation Label Information		
Flower Stages	2mm bud	253
	4mm bud	669
	6mm bud	696
	8mm flower	313
	12mm flower	296
Fruit Stages	nascent	588
	mini	266
	unripe	943
	semi ripe	354
	fully ripe	2,135

**(a)** TomatoMAP-Cls and TomatoMAP-Det label information. **(b)** TomatoMAP-Seg label information. BBCH scale^36,37^ is used for fine-grained stage classification. The BBCH index^36,37^ table can be found in Supplementary Table 1.

### BBCH-Based Classification

The BBCH scale^36,37^ provides a standardized and widely applicable framework for describing the phenological development of plants, encompassing all stages from germination to senescence. Supplementary Table 1 shows the BBCH scale^36,37^ used for *S. lycopersicum*. This scale facilitates precise communication in agronomic research, supports the formulation of crop management strategies, and guides the timing of agrochemical interventions. The BBCH scale^36,37^ is adopted as the primary framework for class-label assignment, providing a systematic and biologically consistent representation of developmental stages. Progression between BBCH stages^36,37^ does not necessarily require the complete cessation of the preceding stage; rather, stages may overlap temporally. Features shared across multiple stages should therefore not be interpreted as redundant. Instead, their recurrence enables the model to learn phenological trajectories and to incorporate temporal dynamics of trait expression. This developmental continuity underscores the need for temporally aware classification strategies. The final distribution of the 50 annotated BBCH classes^36,37^ for key phenological stages of *S. lycopersicum* is presented in Supplementary Table 2.

### Progressive Labeling Workflow for Object Detection

A progressive, AI-assisted annotation protocol is implemented to efficiently label the TomatoMAP-Det subset. The workflow began with a manual labeling phase in Label Studio^40^, which is applied to an initial subset of 1,780 images captured from different camera perspectives. This manually annotated dataset is used to train a first assistive deep learning model. That model is then deployed to produce preliminary labels for an additional 2,504 images; all predictions are rigorously reviewed and corrected by domain experts and merged with the initial set to train a second assistive model. The second model is applied to a further subset drawn from a larger pool of 6,000 images, after which manual expert validation is again performed. The final assistive model, representing the third iteration, is trained using the cumulative annotations from all previous subsets. As a final quality-control step, all annotation files are cross-checked by five experts. At every phase, the image subsets are balanced across camera perspectives to ensure representation consistency.

To ensure high-quality annotations, a rigorous quality-control protocol is implemented. Bounding boxes tightly enclose each target object. For partially visible objects, annotators draw boxes that cover all visible regions together with the morphologically plausible occluded extent, providing consistent representations under varying degrees of occlusion. Objects with extremely limited visible regions, where the structural outline cannot be reliably inferred, are left unannotated, even if they are observable from other viewpoints.

Given the structural complexity of plants, overlap between different classes is permitted, whereas intra-class overlap exceeding 70% is prohibited to avoid redundancy. Adjacent independent objects are individually annotated to maintain granularity. Annotation consistency is maintained across the entire dataset through a three-step verification process: self-inspection by annotators covering all samples, random sampling review of at least 10% of annotations by senior annotators, and cross-validation among different annotators to minimize subjective bias and ensure inter-annotator agreement.

All annotations strictly adhere to predefined class taxonomies. They are defined according to botanical principles in consultation with domain experts.

**Fig. 3:**
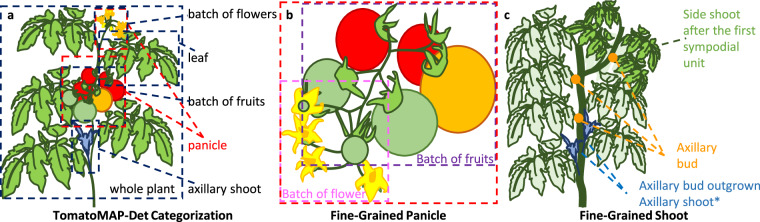
TomatoMap-Det annotation protocol. **(a)** TomatoMAP-Det categorization. Different ROIs are labeled using rectangular bounding boxes. It’s important to note that certain classes may have overlapping relationships. The pot is excluded from the “whole plant”. **(b)** Fine-grained panicle. The panicle class encompasses the flower cluster class and the fruit cluster class. The bounding box can overlap in the situation when multiple clusters of flower or fruit grow on the same panicle. **(c)** Fine-grained shoot phenotypes, including the difference of an axillary shoot, an axillary bud, and a side shoot. Axillary buds have the potential to develop into new shoots, maturing into either axillary shoot or a flower cluster, marked in figure with (*).

For fine-grained object detection, the annotation mainly focused on seven biologically relevant ROIs: whole plant, leaf, panicle, flower clusters, fruit clusters, shoot, and axillary shoot. The biological definitions are shown in Fig. 3. Significantly, the axillary shoot grows out of an axillary bud, which also has the potential to develop a flower and fruit clusters. Given the structural complexity of tomato plants, especially in the context of overlapping reproductive structures, annotations for panicle-related classes may involve intersecting regions when multiple fruit or flower clusters coexist within the same inflorescence.

### Annotation for Fine-Grained Semantic and Instance Segmentation

To facilitate the development of high accuracy model for fine-grained semantic and instance segmentation, a comprehensive annotation protocol is implemented. This protocol involves the labeling of TomatoMAP-Seg, using pixel-wise masks. The annotation process is structured according to the semantic index depicted in Supplementary Fig. 2. This index is referenced from the developmental stages of *S. lycopersicum* flowers and fruits^41^.

The semantic classes for floral structures are defined based on length measurements, with categories corresponding to 2 mm, 4 mm, 6 mm, 8 mm, and 12 mm. The fruit development stages are categorized based on colorimetric and morphological criteria, comprising five classes: nascent, mini, unripe, semi-ripe, and fully ripe. Following these criteria, the domain experts have been engaged in the interactive annotation workflow that integrates automated segmentation proposals with manual refinement to ensure high labeling fidelity across TomatoMAP-Seg. The annotation is performed using the Interactive Semi-Automatic Annotation Tool (ISAT)^42^ with Segment Anything Model 2 (SAM2)^43^.

**Fig. 4:**
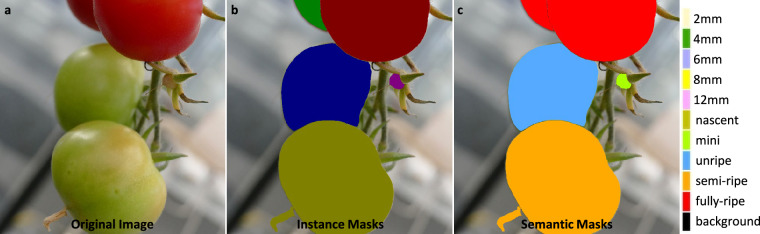
TomatoMap-Seg annotation. Semantic and instance masks showing in different colors following the semantic protocol. A pixel-wise labeling process is proposed. Masks are also labeled by instance IDs and class IDs. **(a)** The original image. **(b)** Visualization of labeled instance segmentation masks, each individual color references an individual instance. **(c)** Visualization of labeled semantic segmentation masks, same color instances belong to same class.

An overview of the annotation workflow is shown in Fig. 4. Each labeled instance includes pixel-wise segmentation masks and unique instance identifiers, supporting both semantic and instance segmentation tasks.

### Cascading Structure

**Fig. 5:**
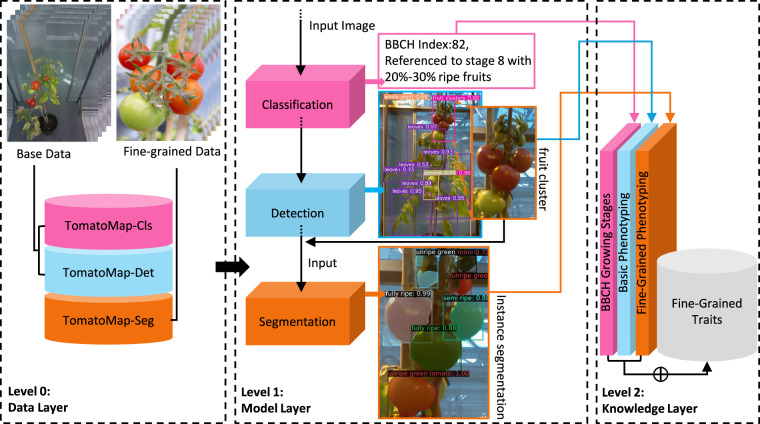
TomatoMAP cascading structure. Level 0 shows the upstream cascading data structure. In detail, TomatoMAP-Cls is the subset for classification, TomatoMAP-Det is the subset for detection, and TomatoMAP-Seg for instance and semantic segmentation. Level 1 shows the streamline of cascading model structure. A progressive specialization strategy is introduced, employing increasingly refined models. The input for segmentation model is cascaded with the output from detection model, the detection model select the ROI from interested plants’ growth stages based on the BBCH index classified by classification model. Finally, all the knowledge forms the final fine-grained phenotypic traits at level 2.

A three-level cascading structure is proposed to support efficient fine-grained phenotyping, comprising data layer cascading (Level 0), model layer cascading (Level 1), and knowledge layer cascading (Level 2). At the data layer, TomatoMAP is structured in a sequential pipeline. Data are progressively refined according to the granularity requirements and task specifications at each stage. At the model layer, a progressive specialization strategy is introduced, whereby increasingly refined models are employed to enhance specificity and efficiency. This design enables effective resource utilization and facilitates parallel processing of multiple images. At the knowledge layer, comprehensive fine-grained phenotypic data are integrated and centrally validated. The overall structure is depicted in Fig. 5. The cascading strategy mitigates performance degradation across the phenotyping pipeline by limiting error propagation between stages^44^. Furthermore, the modular structure allows each component to be validated independently, thereby improving robustness and maintainability.

### FAIR Principles and Domain Shift Analysis

In contrast to many current efforts that focus primarily on scholar-centered data access, the FAIR guiding principles^45^ underscore the importance of machine-friendly data, allowing automated discovery, access, interoperability, and reuse. Such capabilities are essential in our cascading structure, where layered processing is highly dependent on efficient data flow and reusability. Accordingly, the dataset is designed in alignment with these principles to support the requirements of automated, cascading workflows and to maximize its utility within such environments.

To ensure full experimental reproducibility, TomatoMAP-Cls (classification), TomatoMAP-Det (detection), and TomatoMAP-Seg (segmentation) are partitioned into training (70%), validation (20%), and test (10%) subsets using a fixed random seed. For TomatoMAP-Cls, stratified sampling at the class level is employed prior to random selection, yielding 45,099 training, 12,870 validation, and 6,495 test images. TomatoMAP-Det is split using simple random mixed sampling, resulting in 45,124 training, 12,892 validation, and 6,448 test samples. TomatoMAP-Seg, which currently contains 727 annotations, is proportionally divided into 508 training, 146 validation, and 73 test samples. To mitigate class and stage imbalance during dataset construction, images are acquired across diverse developmental stages.

**Fig. 6:**
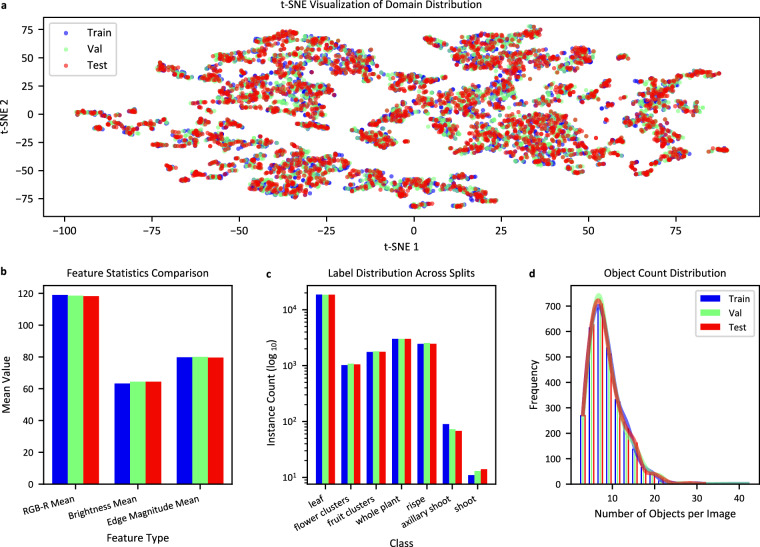
Domain shift analysis across 3,000 standardized samples from training, validation, and test splits with fixed random seed. **(a)** t-SNE visualization of feature space distribution showing the relationship between train (blue), validation (green), and test (red) splits (perplexity: 30, max optimization iteration: 1,000). **(b)** Statistical comparison of selected examples of visual features (RGB-R mean, brightness mean, edge magnitude mean) across splits. **(c)** Class instance distribution on logarithmic scale, showing relative frequencies of all seven tomato plant classes, as well as their balance across spilts. **(d)** Distribution of objects per image across splits.

To evaluate the balance of the resulting splits, 3,000 images are randomly sampled from each subset and a t-SNE based domain distribution analysis is conducted over color (RGB), brightness, edge statistics, texture, frequency-domain descriptors, label distributions, and object-count distributions, shown as Fig. 6.

In Fig. 6a, the t-SNE visualization demonstrates substantial overlap among the three splits, indicating minimal domain shift and consistent data distribution. Fig. 6b shows the statistical analysis of visual features reveals comparable values for RGB-R, brightness, and edge magnitude across all splits, confirming uniform image characteristics. Fig. 6c presents a balanced representation of all seven tomato plant organ classes across splits, despite an overall class imbalance. The object count distribution exhibits nearly identical patterns across splits, as shown in Fig. 6d.

## Data Records

Dataset is deposited in e!DAL (electronic data archive library) of IPK (Leibniz Institute of Plant Genetics and Crop Plant Research): 10.5447/ipk/2025/14^46^. The repository contains two main folders:

- “metadata” contains supporting information of TomatoMAP, including comprehensive manual and semi-automatic phenotypic data for 101 tomato plant samples as well as hardware meta data in CSV files. The dataset includes growth measurements, developmental stage tracking, imaging metadata, and related information.
- “TomatoMAP” folder contains the image data and annotations. The image data is organized into time-series groups. We recommend using our preprocessing scripts to construct the classification and detection datasets. Segmentation annotations can be found directly under the subfolder “TomatoMAP-Seg”.

## Technical Validation

TomatoMAP is validated across three computer vision tasks: fine-grained BBCH-based phenological stage classification^36,37^, multi-object detection for ROI localization, and instance segmentation for precise pixel-level delineation.

Given the generic cascading structure described in section Cascading Structure, models balancing high accuracy with computational efficiency are prioritized to enable real-time inference, a primary objective for TomatoMAP. Accordingly, the stage-wise selections are as follows: for BBCH-based classification, MobileNetv3 Large is employed^47,48^ as a lightweight CNN backbone optimized for efficient and fast feature extraction. For object detection, YOLOv11 Large is adopted^49^, leveraging the CSPDarknet backbone^50^ for efficient multi-scale feature extraction. For instance segmentation, Mask R-CNN with a ResNet-50 backbone and Feature Pyramid Network (FPN) is used^51–53^ to achieve precise pixel-level delineation via deep hierarchical features. To support alternative application objectives, evaluation results for additional classification, detection, and segmentation models, as well as reference results using an inverse-frequency data-balancing method, are provided in the Supplementary Information 7 (see also Fig. 5).

**Fig. 7:**
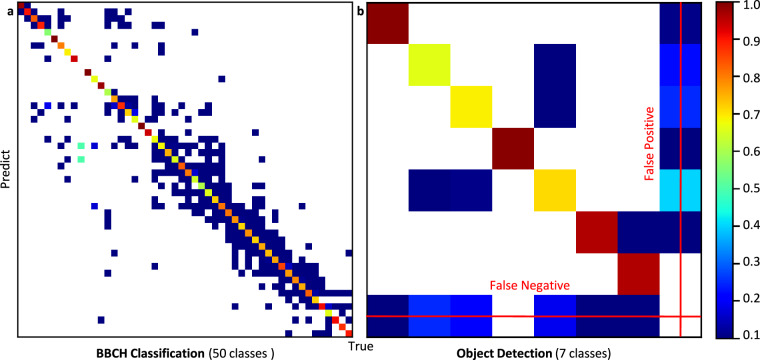
Normalized confusion matrix from MobileNetv3^47,48^ and YOLOv11 Large^49^ on TomatoMAP-Cls and TomatoMAP-Det. **(a)** Validation of BBCH classification for MobileNetv3 Large model^47,48^ with 50 classes^36,37^. Since the BBCH scale^36,37^ is originally designed as a general plant growth stage index, for some plants, certain stages may be skipped. Consequently, the BBCH index^36,37^ shown in this figure does not exhibit a linear relationship with phenological distances between growth stages, since TomatoMAP-Cls includes only a partial representation of the original BBCH scale^36,37^, for example the seedling stage. Based on the data annotation, where only 50 indexes are used, index jumping can appear. White areas inside the matrix refer to 0. Error fluctuation occurs due to the similarities between classes. **(b)** Validation of object detection for fine-tuned YOLOv11 Large^49^ for seven classes. By column normalization, the last bottom row shows the false negative detections, and the last right column shows the false positive detections.

To evaluate the model performance, normalized confusion matrices are generated for both classification and detection tasks, using the validation subsets of TomatoMAP-Cls and TomatoMAP-Det (see Fig. 7). The hyperparameter fine-tuning for YOLOv11 Large^49^ and technical details are shown in Supplementary Information 8. The matrices provide a visible quantitative evaluation of the model’s ability to classify between BBCH-based phenological stages and to detect multiple ROIs.

In Fig. 7a, class imbalance and subtle differences between classes lead to misclassifications that appear near the diagonal, reflecting the biological complexity and visual similarity of adjacent BBCH stages. A manual classification of such stages is labor-intensive and prone to error. Fig. 7b shows strong diagonal dominance in the detection results, particularly for the leaf, whole plant, and shoot categories. However, classes such as flower and fruit clusters, as well as panicles, show elevated rates of false positives and false negatives, indicating areas requiring further refinement.

**Fig. 8:**
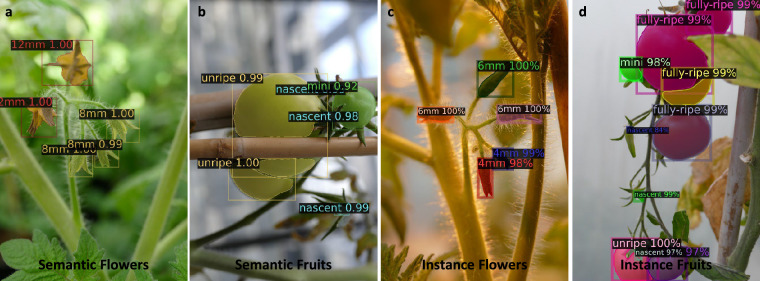
Examples from the prediction results with 0.5 confidence under different scenes. Sub-figures **(a)** and **(b)** display semantic segmentation results for clusters of flower and fruit, respectively, with the class name and confidence labeled at the top-left of each bounding box. Items of the same mask color belong to the same semantic category. Sub-figures **(c)** and **(d)** present instance segmentation results, where individual flowers and fruits are masked in different colors. The confidence score for each instance is displayed in the top-left corner of its corresponding bounding box.

Figure 8 visualizes segmentation outcomes at the flowering and fruiting stages: Subfigures (a) and (b) present semantic segmentation results. Items of the same mask color belong to the same semantic category. Subfigures (c) and (d) present instance segmentation outputs. Each instance is color-coded individually, with its confidence score and category label displayed in the top-left corner of the bounding box. The model performance is particularly strong for unripe, semi ripe and fully ripe fruits, as well as flowers exceeding 4 mm in length. In contrast, the performance declines for early-stage classes such as nascent fruits and 2 mm buds, highlighting the challenge of detecting smaller, low-contrast objects.

Overall, these results highlight TomatoMAP-Seg’s effectiveness in capturing fine-grained morphological traits throughout reproductive development, supporting its utility for downstream phenotypic analysis in plant research.

To quantitatively evaluate the inter-rater agreement^54^ between human annotators and AI, two complementary methods are employed: Cohen’s Kappa^55^ and inter-rater agreement heatmaps^54^. Cohen’s Kappa^55^ is utilized to measure the statistical agreement between pairs of annotators, while inter-rater agreement heatmaps^54^ provide an intuitive visualization of spatial annotation consistency across the pixel domain (see Supplementary Information 3).

Cohen’s Kappa^55^ is calculated for each pairwise combination of annotators, including comparisons between human experts and AI model, where *P*_*o*_ is the observed agreement and *P*_*e*_ is the expected agreement due to chance. The kappa score is defined as:

1 $$\kappa =\frac{{P}_{o}-{P}_{e}}{1-{P}_{e}},$$

**Fig. 9:**
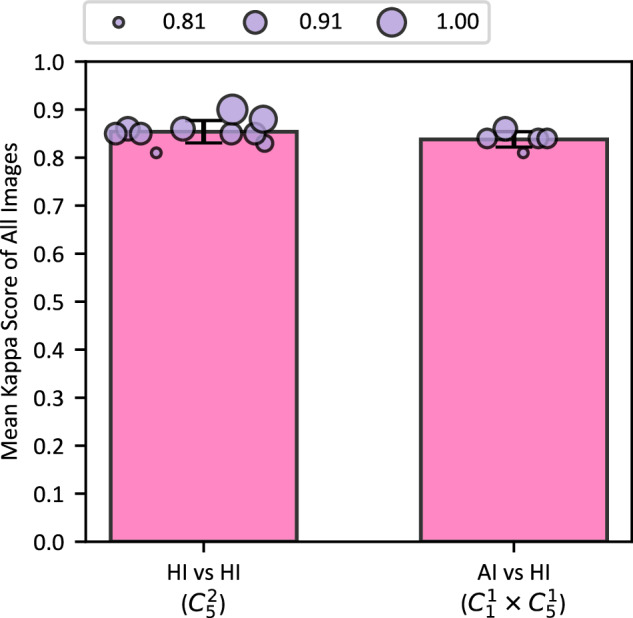
Comparison between human and an AI model trained with TomatoMAP. Mean Cohen’s kappa score for all images for each experimental pair. By calculating the Cohen’s kappa between five domain experts (HI vs HI, ${C}_{5}^{2}$) and between AI model and each expert (AI vs HI, ${C}_{1}^{1}\times {C}_{5}^{1}$) for 295 images external to TomatoMAP, we feature the mean Cohen’s kappa based on each experiment pair. The “HI vs HI” group exhibit “almost perfect agreement” between experts, while the “AI vs HI” group shows the same agreement.

The value of kappa score ranges from -1 (complete disagreement) to 1 (perfect agreement), where 0 indicating no better agreement than random chance. The interpretation of the *κ* values follows established guidelines^56,57^, as detailed in Supplementary Table 6. The analysis pipeline starts with pre-processing and deduplication. Each annotator’s YOLO-format labels are preprocessed first by iterating over all the bounding boxes. Highly overlapping bounding boxes of semantically related classes (e.g. flower clusters, fruit clusters, and panicle) are merged to reduce inter-rater redundancy. To accommodate minor spatial discrepancies in object localization and to associate bounding boxes produced by different annotators, for example, slight positional offsets for the same semantic instance, an Intersection-over-Union (IoU) based matching algorithm is employed. Box pairs are matched only if their IoU threshold *θ* exceeds a given threshold, e.g. *θ* ≥ 0.5. Then Cohen’s Kappa^55^ is computed between five domain experts for human intelligence (HI) comparison (HI vs HI, ${C}_{5}^{2}$) and between AI model and each expert (AI vs HI, ${C}_{1}^{1}\times {C}_{5}^{1}$) for 295 images external to TomatoMAP. Results are shown in Fig. 9.

Based on Cohen’s Kappa coefficient^56,57^, “HI vs HI” groups exhibit “almost perfect agreement” between experts, while the “AI vs HI” group shows the same agreement. The inter-rater agreement heatmaps (see Supplementary Fig. 1) further demonstrated the concordance of human annotations in regions of biological relevance. Moderate disagreement in peripheral regions is likely attributable to subjectivity in boundary delineation, particularly in the absence of clear anatomical landmarks. Without importing stochastic layers, augmentation, and other random parameters, YOLOv11^49^ model trained with TomatoMAP demonstrated high internal consistency across inference replicates, offering a significant advantage over human annotators, whose performance typically exhibits variability.

The result proves the high alignment of AI and human in phenotyping task and underscore the potential of AI-assisted phenotyping in eliminating bias, reducing the time and labor costs of manual phenotyping.

## Limitations

Owing to intrinsic biological characteristics of tomato plants, specifically the lower prevalence of flower and fruit clusters relative to leaves, achieving balanced annotations and AI-human performance comparison across organ classes are challenging. Although the inverse frequency weighting algorithm improves overall model performance, certain classes remain undertrained. Moreover, within the proposed cascading structure, practical criteria for selecting models that optimally balance performance against constraints of real time deployment remain insufficiently established. Future research will focus on developing models constrained by GFLOPs and computational resources of individual edge devices, while enhancing parallel processing efficiency and obtaining high-quality fine-grained phenotypic data.

## Supplementary information

Supplementary information

## Data Availability

The TomatoMAP dataset described in this Data Descriptor is publicly available through the e!DAL (electronic data archive library) of the IPK (Leibniz Institute of Plant Genetics and Crop Plant Research), associated with the following 10.5447/ipk/2025/14.

## Code availability

The scripts for constructing TomatoMAP-Cls and TomatoMAP-Det, as well as the code used for model evaluation, are available at: https://github.com/0YJ/TomatoMAP. The code contains two main functions: TomatoMAP_builder.ipynb offers a step-by-step workflow for processing the raw data into training-ready datasets. The main.py serves as the unified entry point for multiple models, facilitating the training, evaluation, and visualization of classification, detection, and segmentation tasks. The intrinsic matrices and distortion coefficients for our camera modules are stored in .npz format under the repository.

## References

[1] Gerszberg, A., Hnatuszko-Konka, K., Kowalczyk, T. & Kononowicz, A. K. Tomato (Solanum lycopersicum L.) in the service of biotechnology. *Plant Cell Tiss Organ Cult* **120**, 881–902, 10.1007/s11240-014-0664-4 (2015).

[2] Liu, W. *et al*. Solanum lycopersicum, a model plant for the studies in developmental biology, stress biology and food science. *Foods* **11**, 2402, 10.3390/foods11162402 (2022). 36010400 10.3390/foods11162402 PMC9407197

[3] European Commission, Eurostat. Production of tomatoes for fresh consumption. *European Commission* https://agridata.ec.europa.eu/extensions/DashboardFruitAndVeg/FruitandVegetableProduction.html (Accessed 28 April 2025).

[4] Kimura, S. & Sinha, N. Tomato (Solanum lycopersicum): A Model Fruit-Bearing Crop. *CSH Protocols* **2008**, pdb.emo105 10.1101/pdb.emo105 (2008). 10.1101/pdb.emo105 21356708

[5] Aflitos, S. *et al*. Exploring genetic variation in the tomato (Solanum section Lycopersicon) clade by whole-genome sequencing. *The Plant Journal* **80**, 136–148, 10.1111/tpj.12616 (2014). 25039268 10.1111/tpj.12616

[6] Cobb, J. N., Declerck, G., Greenberg, A., Clark, R. & McCouch, S. Next-generation phenotyping: requirements and strategies for enhancing our understanding of genotype-phenotype relationships and its relevance to crop improvement. *Theor Appl Genet* **126**, 867–887, 10.1007/s00122-013-2066-0 (2013). 23471459 10.1007/s00122-013-2066-0 PMC3607725

[7] Singh, M. *et al*. Enhancing genetic gains through marker-assisted recurrent selection: from phenotyping to genotyping. *CEREAL RESEARCH COMMUNICATIONS* **50**, 523–538, 10.1007/s42976-021-00207-4 (2022).

[8] Dong, Q., Louarn, G., Wang, Y., Barczi, J.-F. & de Reffye, P. Does the structure-function model GREENLAB deal with crop phenotypic plasticity induced by plant spacing? A case study on tomato. *Annals of Botany* **101**, 1195–1206, 10.1093/aob/mcm317 (2008). 18199575 10.1093/aob/mcm317 PMC2710282

[9] Schmitz, G. & Theres, K. Genetic control of branching in Arabidopsis and tomato. *Current Opinion in Plant Biology* **2**, 51–55, 10.1016/S1369-5266(99)80010-7 (1999). 10047573 10.1016/s1369-5266(99)80010-7

[10] Jana, S. & Parekh, R. Shape-based Fruit Recognition and Classification. In *Computational Intelligence, Communications, and Business Analytics* (eds Mandal, J. K., Dutta, P. & Mukhopadhyay, S.) *Communications in Computer and Information Science* **776**, 184–196 (Springer Singapore, 2017).

[11] Nithya, S., Sethuraman, O. S. & Sasikumar, K. Effect of Vermicompost and Organic Fertilizer on Improved Growth, Productivity and Quality of Tomato (Solanum lycopersicum) Plant. *Indian Journal of Science and Technology* **17**, 142–148, 10.17485/IJST/v17i2.1821 (2024).

[12] Blanchard-Gros, R. *et al*. Comparison of Drought and Heat Resistance Strategies among Six Populations of Solanum chilense and Two Cultivars of Solanum lycopersicum. *Plants* **10**, 1720, 10.3390/plants10081720 (2021). 34451764 10.3390/plants10081720 PMC8398976

[13] Song, P., Wang, J., Guo, X., Yang, W. & Zhao, C. High-throughput phenotyping: Breaking through the bottleneck in future crop breeding. *The Crop Journal* **9**, 633–645, 10.1016/j.cj.2021.03.015 (2021).

[14] Tian, S., Fang, C., Zheng, X. & Liu, J. Lightweight detection method for real-time monitoring tomato growth based on improved YOLOv5s. *IEEE Access* **12**, 29891–29899, 10.1109/ACCESS.2024.3368914 (2024).

[15] Lee, U. *et al*. An automated, clip-type, small Internet of Things camera-based tomato flower and fruit monitoring and harvest prediction system. *Sensors* **22**, 2456, 10.3390/s22072456 (2022). 35408071 10.3390/s22072456 PMC9002604

[16] Rahman, F. A. *et al*. Growth monitoring of greenhouse tomatoes based on context recognition. *AgriEngineering* **6**, 2043–2056, 10.3390/agriengineering6030119 (2024).

[17] Islam, M. P. & Hatou, K. Artificial intelligence assisted tomato plant monitoring system—an experimental approach based on universal multi-branch general-purpose convolutional neural network. *Computers and Electronics in Agriculture* **224**, 109201, 10.1016/j.compag.2024.109201 (2024).

[18] Baar, S. *et al*. A logistic model for precise tomato fruit-growth prediction based on diameter-time evolution. *Computers and Electronics in Agriculture* **227**, 109500, 10.1016/j.compag.2024.109500 (2024).

[19] Bar, M. & Ori, N. Compound leaf development in model plant species. *Current Opinion in Plant Biology* **23**, 61–69, 10.1016/j.pbi.2014.10.007 (2015). 25449728 10.1016/j.pbi.2014.10.007

[20] Zhu, Y. *et al*. Quantitative Extraction and Evaluation of Tomato Fruit Phenotypes Based on Image Recognition. *Frontiers in Plant Science* **13**, 859290, 10.3389/fpls.2022.859290 (2022). 35498696 10.3389/fpls.2022.859290 PMC9044966

[21] Gehlot, M., Saxena, R. K. & Gandhi, G. C. “Tomato-Village”: a dataset for end-to-end tomato disease detection in a real-world environment. *Multimedia Systems* **29**, 3305–3328, 10.1007/s00530-023-01158-y (2023).

[22] Gehlot, M., Saxena, R. K. & Gandhi, G. C. “Tomato-Village”: a dataset for end-to-end tomato disease detection in a real-world environment. *Github* https://github.com/mamta-joshi-gehlot/Tomato-Village.

[23] Tsironis, V., Bourou, S. & Stentoumis, C. tomatOD: Evaluation of Object Detection Algorithms on a New Real-World Tomato Dataset. journalISPRS - International Archives of the Photogrammetry, Remote Sensing and Spatial Information Sciences, 1077–1084 10.5194/isprs-archives-XLIII-B3-2020-1077-2020 (2020).

[24] Tsironis, V., Bourou, S. & Stentoumis, C. tomatOD: Evaluation of object detection algorithms on a new real-world tomato dataset. *Github* https://github.com/up2metric/tomatOD.

[25] Hughes, D. P. & Salathé, M. An open access repository of images on plant health to enable the development of mobile disease diagnostics through machine learning and crowdsourcing. *arXiv* preprint https://arxiv.org/abs/1511.08060 (2015).

[26] Mohanty, S. P., Hughes, D. P. & Salathé, M. Using deep learning for image-based plant disease detection. *Frontiers in Plant Science* **7**10.3389/fpls.2016.01419 (2016). 10.3389/fpls.2016.01419 PMC5032846 27713752

[27] Mohanty, S. P., Hughes, D. P. & Salathé, M. Using deep learning for image-based plant disease detection. *Github* https://github.com/spMohanty/PlantVillage-Dataset. 10.3389/fpls.2016.01419 PMC5032846 27713752

[28] Trigubenko, R. & hfujihara. LaboroTomato: Instance segmentation dataset *Github* https://github.com/laboroai/LaboroTomato (2020).

[29] Tian, K. *et al*. Tomato leaf diseases recognition based on deep convolutional neural networks. *Journal of Agricultural Engineering* **54**, 1, 10.4081/jae.2022.1432 (2022).

[30] Tian, K. Tomato leaf image dataset. *Mendeley Data*10.17632/369cky7n39.1 (2020).

[31] Huang, M.-L. & Chang, Y.-H. Dataset of Tomato Leaves. *Mendeley Data*10.17632/ngdgg79rzb.1 (2020).

[32] Khan, S. *et al*. Tomato Leaf Dataset. *Mendeley Data*10.17632/rv3kxfv47y.1 (2020).

[33] Li, X., Xu, L., Zheng, C., Fu, X. Multi-view 3D reconstruction based on SFM and improved deep network. *2022 4th International Conference on Frontiers Technology of Information and Computer (ICFTIC)* 154–159, 10.1109/ICFTIC57696.2022.10075204 (2022).

[34] Liao, G. *et al*. CLIP-GS: CLIP-Informed Gaussian Splatting for Real-time and View-consistent 3D Semantic Understanding. *ACM Transactions on Multimedia Computing, Communications and Applications*, 1–24, 10.1145/3746284 (2025).

[35] Costa, C., Schurr, U., Loreto, F., Menesatti, P. & Carpentier, S. Plant Phenotyping Research Trends, a Science Mapping Approach. *Frontiers in Plant Science* **9**, 1933, 10.3389/fpls.2018.01933 (2018). 30666264 10.3389/fpls.2018.01933 PMC6330294

[36] Feller, C. *et al*. Phänologische Entwicklungsstadien von Gemüsepflanzen II. Fruchtgemüse und Hülsenfrüchte: Codierung und Beschreibung nach der erweiterten BBCH-Skala - mit Abbildungen. *Heft 9* **47**, 217–232 (1995).

[37] Meier, U. *et al*. The BBCH system to coding the phenological growth stages of plants — history and publications. *Kulturpflanzen* **61**, 41–52, 10.5073/JfK.2009.02.01 (2009).

[38] Sun, J. *et al*. Detection of tomato organs based on convolutional neural network under the overlap and occlusion backgrounds. *Machine Vision and Applications* **31**, 31, 10.1007/s00138-020-01081-6 (2020).

[39] Bradski, G. The OpenCV Library. *Dr. Dobb’s Journal of Software Tools*. Available at: https://opencv.org.

[40] Tkachenko, M., Malyuk, M., Holmanyuk, A. & Liubimov, N. Label Studio: Data labeling software. *Github* https://github.com/HumanSignal/label-studio (2020–2025).

[41] Dingley, A. *et al*. Precision pollination strategies for advancing horticultural tomato crop production. *Agronomy* **12**, 518, 10.3390/agronomy12020518 (2022).

[42] Ji, S. & Zhang, H. ISAT with Segment Anything: An Interactive Semi-Automatic Annotation Tool. *Github* https://github.com/yatengLG/ISAT_with_segment_anything (2024).

[43] Ravi, N. *et al*. SAM 2: Segment Anything in Images and Videos. In* International Conference on Learning Representations (ICLR2025)* https://openreview.net/forum?id=Ha6RTeWMd0 (2025).

[44] Sambasivan, N. *et al*. “Everyone wants to do the model work, not the data work”: Data cascades in high-stakes AI. *Association for Computing Machinery* **39**, 1–15, 10.1145/3411764.3445518 (2021).

[45] Wilkinson, M. D. *et al*. The FAIR Guiding Principles for scientific data management and stewardship. *Sci Data* **3**, 160018, 10.1038/sdata.2016.18 (2016). 10.1038/sdata.2016.18 PMC4792175 26978244

[46] Zhang, Y. *et al*. Tomato Multi-Angle Multi-Pose Dataset for Fine-Grained Phenotyping. *eIDAL*10.5447/ipk/2025/14 (2025). 10.1038/s41597-026-06926-9 PMC12954114 41764239

[47] Howard, A. *et al*. Searching for MobileNetV3. *2019 IEEE/CVF International Conference on Computer Vision (ICCV)*, 1314–1324, 10.1109/ICCV.2019.00140 (2019).

[48] Howard, A. G. *et al*. MobileNets: Efficient Convolutional Neural Networks for Mobile Vision Applications. *arXiv* preprint http://arxiv.org/abs/1704.04861 (2017).

[49] Khanam, R. & Hussain, M. YOLOv11: An overview of the key architectural enhancements. *arXiv* preprint https://arxiv.org/abs/2410.17725 (2024).

[50] Wang, C.-Y. *et al*. CSPNet: A new backbone that can enhance learning capability of CNN. *2020 IEEE/CVF Conference on Computer Vision and Pattern Recognition Workshops (CVPRW)*, 1571–1580, 10.1109/CVPRW50498.2020.00203 (2020).

[51] He, K., Gkioxari, G., Dollár, P. & Girshick, R. Mask R-CNN. In *Proceedings of the IEEE international conference on computer vision*, 2961–2969, 10.1109/ICCV.2017.322 (2017).

[52] Koonce, B. ResNet 50. In: *Convolutional Neural Networks with Swift for Tensorflow*. Apress, Berkeley, CA, 63–72, 10.1007/978-1-4842-6168-2_6 (2021).

[53] Wu, Y., Kirillov, A., Massa, F., Lo, W.-Y. & Girshick, R. Detectron2 *Github* https://github.com/facebookresearch/detectron2 (2019).

[54] Yang, F. *et al*. Assessing Inter-Annotator Agreement for Medical Image Segmentation. *IEEE Access* **11**, 21300–21312, 10.1109/ACCESS.2023.3249759 (2023). 37008654 10.1109/access.2023.3249759 PMC10062409

[55] Warrens, M. J. Five ways to look at Cohen’s kappa. *J. Psychol. Psychother.* **5**, 1–6, 10.4172/2161-0487.1000197 (2015).

[56] Altman, D. G., Practical statistics for medical research. *Chapman & Hall/CRC*10.1201/9780429258589 (Boca Raton, Fla, 1999).

[57] Landis, J. R. & Koch, G. G. The measurement of observer agreement for categorical data. *Biometrics* 159–174 10.2307/2529310 (1977). 843571

