# Supplementary material for: Tomato Multi-Angle Multi-Pose Dataset for Fine-Grained Phenotyping
Source: Sci Data. 2026 Feb 28;13:309. doi: 10.1038/s41597-026-06926-9 (PMC12954114; doi:10.1038/s41597-026-06926-9)
Supplement: Supplementary file 1 — Supplementary information [file 41597_2026_6926_MOESM1_ESM.pdf]

Supplementary Information

SI.1 Sample Conditioning

To ensure optimal growth of plant samples, the greenhouse environment is controlled to maintain daytime temperatures between 22 ° and 28 °, and nighttime temperatures between 16 ° and 18 °. Relative humidity is regulated within a range of 60–70%. Natural sunlight is supplemented with high-pressure sodium lamps to maintain a consistent 14-hour photoperiod. The plants are grown in containers filled with a stratified soil medium composed primarily of lean clay and silty sand (PATZER ERDEN GmbH, Sinntal-Altengronau, Germany). Irrigation is delivered via a drip system, and plants receive a balanced nutrient solution every 14 days, including a 0.2% foliar application of "WUXAL Basis" (Hauert MANNA Düngerwerke GmbH, Nürnberg, Germany) NPK fertilizer.

SI.2 Data Format

The image data is stored in JPG format and systematically named using the following style: {*pi id*}_{*image id*}_{*plant id*}_{*pose id*}_{*time stamp*}. TomatoMAP-Cls is classified by BBCH folders. The annotation files for TomatoMAP-Det conform to the Ultralytics YOLO format [1], defined by rectangular bounding boxes. Annotations are stored as plain text (.TXT) files, with one row per object instance comprising five parameters: $class\_id, x\_center, y\_center, width, height$. All coordinates are normalized to a $[0, 1]$ range relative to the image dimensions. For TomatoMAP-Seg, the annotation is processed as COCO JSON format [2, 3].

SI.3 Intra-Agreement Heatmap

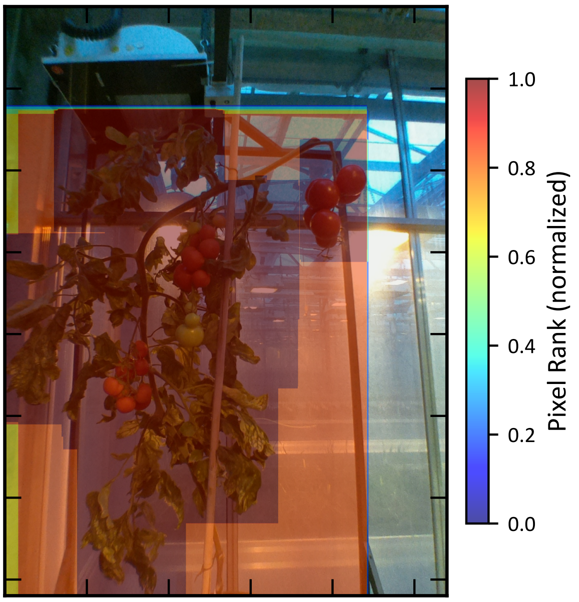

Figure S1: Bias of human annotators. A visualization example of the inter-rater agreement heatmap involving 5 domain experts. Strong agreement is observed in biologically relevant regions, while disagreements emerge at plant boundaries, highlighting human subjectivity in these areas.

In order to visualize the annotation consistency, we generate inter-rater agreement heatmaps [4], which accumulate the bounding boxes from all annotators by rendering them as binary masks and summing the rank by pixel. High-intensity regions on these maps correspond to areas of high inter-rater agreement, while low-intensity regions indicate divergence. This visualization complements the kappa score by providing direct insight into annotation variance. The inter-rater agreement heatmap is generated from 295 additional image data based on their labels [5]. The normalized heatmap value $\text{Heatmap}(x, y) \in [0, 1]$ represents the proportion of annotators whose bounding boxes cover pixel (x, y). This reflects the annotation density at each spatial location and can be used to analyze the spatial consistency of the annotations. For each pixel (x,y), the agreement value is computed as the normalized sum of binary responses across annotators:

$$\text{Heatmap}(x, y) = \frac{1}{N} \sum_{i=1}^{N} \text{BBoxR}_i(x, y) \tag{1}$$

where N is the total number of annotators (in our case, $N = 5$), and $\text{BBoxR}_i(x, y)$ denotes the binary response function of the i-th annotator at pixel location (x, y), defined as:

$$\text{BBoxR}_i(x, y) = \begin{cases} 1, & \text{if the bounding box from annotator } i \text{ covers pixel}(x, y), \\ 0, & \text{otherwise.} \end{cases} \tag{2}$$

SI.4 Index for Semantic Annotation

To establish a comprehensive framework for fine-grained tomato phenotyping and semantics of the developmental stages, we propose a morphometric developmental index that integrates both floral and fruit ontogeny measurements. This index categorizes tomato development into 10 stages based on corresponding morphological characteristics. For flower development, the classification encompasses the progression from initial bud formation (2mm) through intermediate developmental phases (4mm, 6mm, 8mm) to full anthesis with petal expansion and anther dehiscence (12mm). Concurrently, fruit development is divided into five distinct phases: nascent (post-fertilization initiation),

mini (early cell division), unripe (cell expansion with green coloration), semi-ripe (onset of carotenoid accumulation), and fully-ripe (complete lycopene synthesis and red pigmentation). This standardized index provides a robust foundation for semantic segmentation algorithms in our phenotyping pipelines, enabling precise temporal tracking of developmental transitions and facilitating quantitative analysis of growth dynamics in tomato breeding and physiological studies.

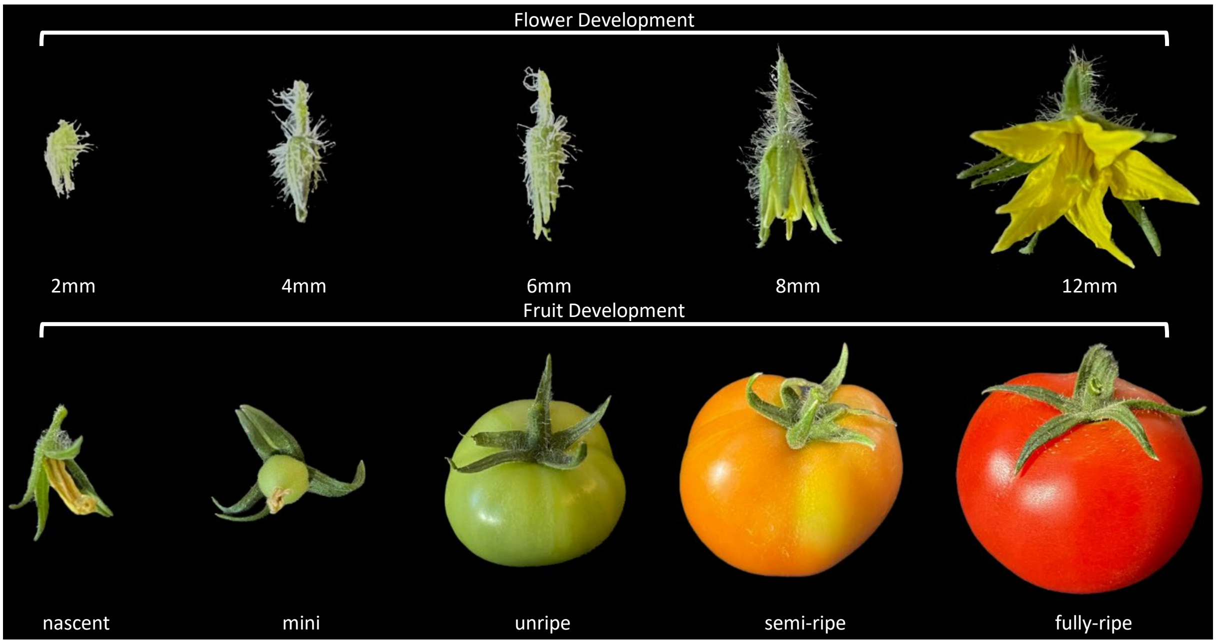

Figure S2: Index for semantic annotation of flowers and fruit of *S. lycopersicum* [6]. Flower development semantic is signified by a corresponding millimeter range. However, since fruit development stages has specific features, its semantic is named directly based on those features. Our semantic and instance segmentation processes strictly follow this protocol.

SI.5 BBCH Index for *S. lycopersicum*

Table S1: BBCH [7, 8] code modified for *S. lycopersicum*. The BCCH-scale [7, 8] has normalized and homologated all elements into a two-digit system ranging from 0 to 99, where the first digit representing the main developmental stage and the second digit indicating the fine-grained stage.

BBCH code for *S. lycopersicum*	Description
Principal growth stage 0: Germination	
0	Dry seed
1	Initiation of seed imbibition
3	Seed imbibition completed
5	Radicle emergence from seed
7	Emergence of hypocotyl with cotyledons from the seed
9	Emergence of cotyledons through soil surface
Principal growth stage 1: Leaf development	
10	Cotyledons fully unfolded
11	First elliptic leaf visible
12	First pair of true leaves visible
13	6 of true leaves visible
14	8 of true leaves visible
15	10 of true leaves visible
16	12 of true leaves visible
17	14 of true leaves visible
18	16 of true leaves visible
19	18 or more of true leaves visible
Principal growth stage 2: Side shoot development	
20	First Side shoot start formed
21	10%-20% Side shoots formed
22	20%-30% Side shoots formed
23	30%-40% Side shoots formed
24	40%-50% Side shoots formed
25	50%-60% Side shoots formed
26	60%-70% Side shoots formed
27	70%-80% Side shoots formed
28	80%-90% Side shoots formed
29	90%-100% Side shoots formed
Principal growth stage 5: Inflorescence emergency	
51	First inflorence visible, visible closed flower bud

Table S1: BBCH [7,8] code modified for *S. lycopersicum*. The BCCH-scale [7,8] has normalized and homologated all elements into a two-digit system ranging from 0 to 99, where the first digit representing the main developmental stage and the second digit indicating the fine-grained stage.

BBCH code for *S. lycopersicum*	Description
52	2 inflorescences visible
53	3 inflorescences visible
54	4 inflorescences visible
55	5 inflorescences visible
56	6 inflorescences visible
57	7 inflorescences visible
58	8 inflorescences visible
59	9 or more inflorescences visible
Principal growth stage 6: Flowering	
60	First flowers open
61	10%-20% of flowers open
62	20%-30% of flowers open
63	30%-40% of flowers open
64	40%-50% of flowers open
65	Full flowering, 50%-60% of flowers open, first petals falling
66	Full flowering, 60%-70% of flowers open, more petals falling
67	Full flowering, 70%-80% of flowers open, more petals falling
68	Full flowering, 80%-90% of flowers open, more petals falling
69	End of flowering, 90%-100% of flowers open, more petals falling
Principal growth stage 7: Fruit development	
70	Fruits at the main stem or branches visibles
71	10%-20% of final fruit size
72	20%-30% of final fruit size
73	30%-40% of final fruit size
74	40%-50% of final fruit size
75	50%-60% of final fruit size
76	60%-70% of final fruit size
77	70%-80% of final fruit size
78	80% -90% of final fruit size
79	90% -100% of final fruit size
Principal growth stage 8: Maturity of fruit	
80	fruits start showing typical ripe color (orange → red)
81	10%-20% of fruits show typical fully ripe color
82	20%-30% of fruits show typical fully ripe color
83	30%-40% of fruits show typical fully ripe color
84	40%-50% of fruits show typical fully ripe color
85	50%-60% of fruits show typical fully ripe color
86	60%-70% of fruits show typical fully ripe color
87	70%-80% of fruits show typical fully ripe color
88	80%-90% of fruits show typical fully ripe color
89	90%-100% of fruits show typical fully ripe color
Principal growth stage 9: Senescence	
97	All leaves fallen
99	Post harvest or storage treatmen

SI.6 BBCH-Based Fine-Grained TomatoMAP-Cls Class Distribution

Table S2: The phenological development stages of *S. lycopersicum* have been standardized using the BBCH index [7,8]. This table displays how various growth stages are distributed across TomatoMAP-Cls.

BBCH Stages	TomatoMAP-Cls Classes
Leaf development	13 14 15 16 17 19
Side shoot development	20 21 22 23 27 28 29
Inflorescence emergency	51 52 53 54 55 56 59
Flowering	60 61 62 63 64 65 66 67 68 69
Fruit development	70 71 72 73 74 75 76 77 78 79
Maturity of fruit	80 81 82 83 84 85 86 87 88 89

SI.7 Implementations on TomatoMAP

To verify the effectiveness of models, ViT Large [9], Swinv2 Large [10], and MobileNetv3 Large [11,12] are compared. All models are fine-tuned from ImageNet-pretrained weights [13] with a batch size of 16, using AdamW optimizer with a learning rate of 0.5×10^{-4} and weight decay of 0.05. The learning rate is decayed by a factor of 0.1 every 30 epochs. Early stopping (patience=3) is applied based on validation accuracy. For inference speed evaluation, we measure FPS with batch size 1 after 10 warmup iterations to stabilize GPU performance. TomatoMAP-Cls achieved an overall test accuracy of 73.39% and inference FPS 151.53 on MobileNetv3 [11,12] with NVIDIA Tesla

V100 PCIe (16 GB) GPU, which demonstrates its efficiency for fine-grained visual phenotyping in our cascading structure, despite the inherent complexity of the classification task. see Table S3.

Table S3: Model Comparison on TomatoMAP-Cls.

Model	Type	Best Epoch	Input Dimension	Test Accuracy	Inference FPS
ViT Large [9]	Vision Transformer	6	224×224	81.34	61.53
Swinv2 Large [10]	Vision Transformer	12	192×192	83.03	36.49
**MobileNetv3 Large [11, 12]**	**CNN**	**40**	**224×224**	**73.39**	**151.53**

To eliminate data imbalance, weighted sampling is employed during training TomatoMAP-Det. Class weights are calculated using inverse frequency [14]:

$$w_i = \frac{\sum_{j=1}^{C} n_j}{n_i} \tag{3}$$

where C is the total number of classes, n_i is the number of instances for class i, and w_i is the weight for class i. For images containing multiple objects, the image-level weight is:

$$W_k = \frac{1}{m_k} \sum_{i=1}^{m_k} w_{c_i} \tag{4}$$

where m_k is the number of objects in image k, and c_i denotes the class index of each object. The sampling probability for each image is:

$$p_k = \frac{W_k}{\sum_{j=1}^{N} W_j} \tag{5}$$

where N is the total number of training images. Images are sampled according to $k \sim \text{Categorical}(p_1, \ldots, p_N)$, ensuring rare classes receive more attention during training. A comparison is performed between CNN based YOLOv11 Large [1], YOLOv11 Nano [1], and RT-DETR Large [15, 16] with hybird CNN-transformer architecture. All models are initialized from COCO pretrained weights [2, 3] with a batch size of 16, using the SGD optimizer (momentum=0.9) with an initial learning rate of 0.01 and weight decay of 5×10^{-4}. The learning rate is decayed using cosine annealing. Early stopping (patience=5) is applied based on validation metrics. Input resolution is 640×640. All experiments are conducted on a NVIDIA A100 PCIe (80 GB) GPU, shown as Table S4

Table S4: Model Comparison on TomatoMAP-Det.

Model	Type	Best Epoch	mAP50	Inference FPS
YOLOv11 Nano [1]	CNN	203	0.61	60.46
YOLOv11 Nano (Weighted) [1, 14]	CNN	160	0.75	69.15
YOLOv11 Large [1]	CNN	167	0.71	51.35
**YOLOv11 Large (Weighted) [1, 14]**	**CNN**	**171**	**0.91**	**49.66**
RT-DETR Large [15, 16]	Hybrid (CNN + Transformer)	22	0.60	30.52
RT-DETR Large (Weighted) [14–16]	Hybrid (CNN + Transformer)	23	0.74	30.28

Three Mask R-CNN configurations are compared: R50-FPN-1×, R50-FPN-3×, and R101-FPN-3× [17–19], all initialized from COCO pretrained weights [2, 3]. 1× and 3× refer to the pretraining schedule on COCO [2, 3]. Models are trained with SGD optimizer (momentum=0.9), a batch size of 4, and using learning rates in $\{1.2, 2.4, 4.8, 9.6\} \times 10^{-4}$ and weight decay 1×10^{-4}. Training runs for up to 100 epochs with validation every 10 epochs and early stopping (patience=5). COCO evaluation protocol [2, 3] with mask AP as the primary metric [17–19] is used. All experiments are conducted on a NVIDIA A100 PCIe (80 GB) GPU, shown as Table S5.

Table S5: Implementations on TomatoMAP-Seg.

Model	Learning Rate (10^{-4})	Best Epoch	Mask AP-50
Mask R-CNN R50-FPN 1x [17–19]	1.20	90	62.69
Mask R-CNN R50-FPN 1x [17–19]	2.40	80	64.00
Mask R-CNN R50-FPN 1x [17–19]	4.80	90	63.56
**Mask R-CNN R50-FPN 1x [17–19]**	**9.60**	**40**	**67.11**
Mask R-CNN R50-FPN 3x [17–19]	1.20	90	64.47
Mask R-CNN R50-FPN 3x [17–19]	2.40	60	65.38
Mask R-CNN R50-FPN 3x [17–19]	4.80	50	64.73
Mask R-CNN R50-FPN 3x [17–19]	9.60	40	64.27
Mask R-CNN R101-FPN 3x [17–19]	1.20	80	63.52
Mask R-CNN R101-FPN 3x [17–19]	2.40	40	64.21
Mask R-CNN R101-FPN 3x [17–19]	4.80	30	65.87
Mask R-CNN R101-FPN 3x [17–19]	9.60	20	64.87

SI.8 Hyperparameter Fine Tuning on YOLOv11 Large

To optimize model performance, a hyperparameter search is performed within predefined parameter spaces. Each configuration is trained for 150 epochs, with fitness evaluated using a weighted hybrid metric combining precision, recall, and mAP scores, shown as Fig. S3.

The final fine-tuned model is trained with the optimal hyperparameters with a batch size of 8 and input resolution of 640 × 640. Training is conducted for 1,200 epochs with early stopping (patience=50) applied based on validation metrics. The best epoch is 1,077 with 0.98 mAP50 and 0.92 mAP50-95. Class balancing is not applied. The SGD optimizer is employed with an initial learning rate of 8.9×10^{-4}, learning rate factor of 0.009, momentum of 0.6, and weight decay of 1.6×10^{-4}, using warmup scheduling over 7.23 epochs. All experiments are conducted on a NVIDIA A100 PCIe (80 GB) GPU.

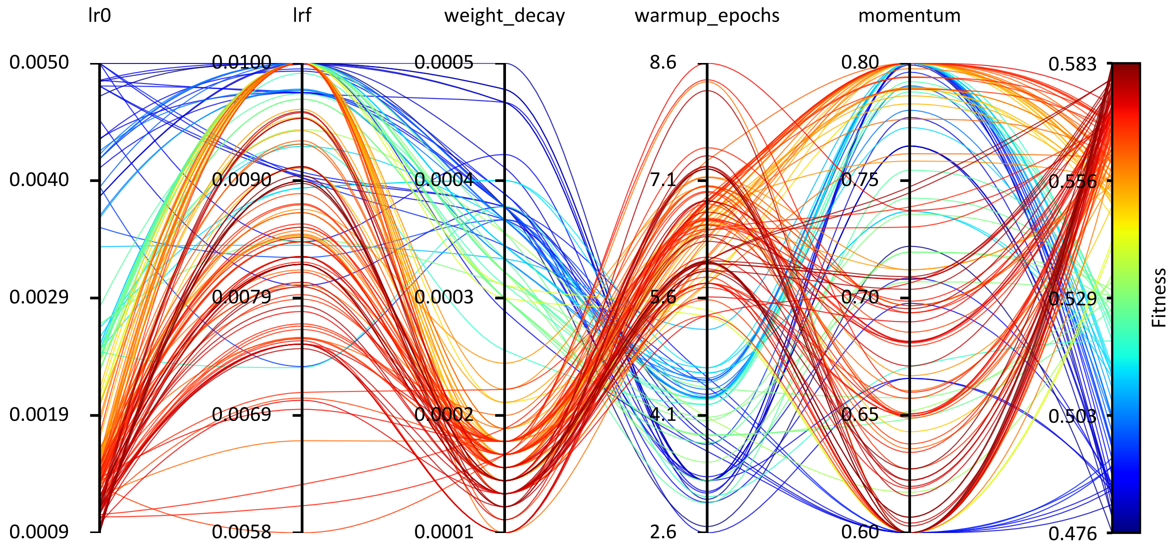

Figure S3: Parallel coordinates for YOLOv11 Large [1] hyperparameter fine tuning on TomatoMAP-Det. To optimize model performance, we initially conduct hyperparameter search within predefined parameter spaces, comparing lr0 (initial learning rate), lrf (learning rate factor), weight_decay (L2 regularization), warmup_epochs (learning rate warm up period), and momentum (gradient accumulation factor). Each configuration is trained for 150 epochs, with fitness evaluated using a weighted hybrid metric combining precision, recall, and mAP scores.

SI.9 Cohen's Kappa Agreement Map

Table S6: Cohen's Kappa scores and their matched agreements based on the pipeline from Altman [20] and improved by Landis et al. [21]

Cohen's Kappa statistic (κ)	Strength of agreement
< 0.20	None to slight agreement
0.21–0.39	Fair agreement
0.40–0.59	Moderate agreement
0.60–0.79	Substantial agreement
0.80–0.90	Almost perfect agreement
> 0.90	Almost perfect agreement

References

[1] Khanam, R. & Hussain, M. YOLOv11: An overview of the key architectural enhancements. *arXiv* preprint, `https://arxiv.org/abs/2410.17725` (2024).

[2] Lin, T.-Y. *et al.* Microsoft COCO: Common Objects in Context. In: Fleet, D., Pajdla, T., Schiele, B., Tuytelaars, T. (eds) *Computer Vision – ECCV 2014. Lecture Notes in Computer Science*, **8693**, `https://doi.org/10.1007/978-3-319-10602-1_48` (2014).

[3] Lin, T.-Y. *et al.* COCO Dataset. `https://cocodataset.org` (2014).

[4] Yang, F. *et al.* Assessing Inter-Annotator Agreement for Medical Image Segmentation. *IEEE Access* **11**, 21300-–21312, `https://doi.org/10.1109/ACCESS.2023.3249759` (2023).

[5] Begley, C. G. & Ioannidis, J. P. A. Reproducibility in science: improving the standard for basic and preclinical research. *Circulation Research* **116**, 116–126, `https://doi.org/10.1161/CIRCRESAHA.114.303819` (2015).

[6] Dingley, A. *et al.* Precision pollination strategies for advancing horticultural tomato crop production. *Agronomy* **12**, 518, `https://doi.org/10.3390/agronomy12020518` (2022).

[7] Feller, C. *et al.* Phänologische Entwicklungsstadien von Gemüsepflanzen II. Fruchtgemüse und Hülsenfrüchte: Codierung und Beschreibung nach der erweiterten BBCH-Skala - mit Abbildungen. *Heft 9* **47**, 217-232, `https://www.openagrar.de/receive/openagrar_mods_00067073` (1995).

[8] Meier, U. *et al.* The BBCH system to coding the phenological growth stages of plants — history and publications. *Kulturpflanzen* **61**, 41–52, `https://doi.org/10.5073/JfK.2009.02.01` (2009).

[9] Dosovitskiy, A. *et al.* An image is worth 16x16 words: Transformers for image recognition at scale. In *International Conference on Learning Representations (ICLR 2021)*, `https://openreview.net/forum?id=YicbFdNTTy` (2021).

[10] Liu, Z. *et al.* Swin Transformer V2: Scaling up capacity and resolution. *2022 IEEE/CVF Conference on Computer Vision and Pattern Recognition (CVPR)*, 11999-12009, `https://doi.org/10.1109/CVPR52688.2022.01170` (2022).

[11] Howard, A. *et al.* Searching for MobileNetV3. *2019 IEEE/CVF International Conference on Computer Vision (ICCV)*, 1314-1324, `https://doi.org/10.1109/ICCV.2019.00140` (2019).

[12] Howard, A. G. *et al.* MobileNets: Efficient Convolutional Neural Networks for Mobile Vision Applications. *arXiv* preprint, `http://arxiv.org/abs/1704.04861` (2017).

[13] Deng, J. *et al.* ImageNet: A large-scale hierarchical image database. In *2009 IEEE Conference on Computer Vision and Pattern Recognition*, 248-–255, `https://doi.org/10.1109/CVPR.2009.5206848` (2009).

[14] Ounis, I. Inverse Document Frequency. In: LIU, L., ÖZSU, M.T. (eds) *Encyclopedia of Database Systems*, 1570–1571, `https://doi.org/10.1007/978-0-387-39940-9_933` (Springer Boston, MA 2009).

[15] Zhao, Y. *et al.* DETRs beat YOLOs on real-time object detection. *2024 IEEE/CVF Conference on Computer Vision and Pattern Recognition (CVPR)*, 16965-16974, `https://doi.org/10.1109/CVPR52733.2024.01605` (2024).

[16] Lv, W. *et al.* RT-DETRv2: Improved baseline with bag-of-freebies for real-time detection transformer. *arXiv* preprint, `https://arxiv.org/abs/2407.17140` (2024).

[17] He, K., Gkioxari, G., Dollár, P. & Girshick, R. Mask R-CNN. In *Proceedings of the IEEE international conference on computer vision,* 2961-2969, `https://doi.org/10.1109/ICCV.2017.322` (2017).

[18] Koonce, B. ResNet 50. In: *Convolutional Neural Networks with Swift for Tensorflow*, 63-–72, `https://doi.org/10.1007/978-1-4842-6168-2_6` (Apress Berkeley, CA, 2021).

[19] Wu, Y., Kirillov, A., Massa, F., Lo, W.-Y. & Girshick, R. Detectron2 *Github*, `https://github.com/facebookresearch/detectron2` (2019).

[20] Altman, D. G. Practical statistics for medical research. *Chapman & Hall/CRC*, `https://doi.org/10.1201/9780429258589` (Boca Raton, Fla, 1999).

[21] Landis, J. R. & Koch, G. G. The measurement of observer agreement for categorical data. *Biometrics*, 159-–174, `https://doi.org/10.2307/2529310` (1977).
